# Sources of variability in quantification of cardiovascular magnetic resonance infarct size - reproducibility among three core laboratories

**DOI:** 10.1186/s12968-017-0378-y

**Published:** 2017-08-11

**Authors:** Igor Klem, Einar Heiberg, Lowie Van Assche, Michele A. Parker, Han W. Kim, John D. Grizzard, Håkan Arheden, Raymond J. Kim

**Affiliations:** 1 Duke Cardiovascular Magnetic Resonance Center, Division of Cardiology, Duke University Medical Center, Durham, USA; 2 Department of Clinical Physiology, Lund University Hospital, Department of Biomedical Engineering, Lund University, Lund, Sweden; 3 Duke Cardiovascular Magnetic Resonance Center, Duke University Medical Center, Durham, USA; 4 Department of Radiology, Virginia Commonwealth University Health Systems, Richmond, USA; 5 Department of Clinical Physiology, Lund University, Lund University Hospital, Lund, Sweden; 6 Duke Cardiovascular Magnetic Resonance Center, Duke South Clinic, Division of Cardiology, Department of Radiology, Duke University Medical Center, Trent Drive, RM 4229, DUMC-3934, Durham, NC 27710 USA

## Abstract

**Background:**

Acute myocardial infarct (AMI) size depicted by late gadolinium enhancement cardiovascular magnetic resonance (CMR) is increasingly used as an efficacy endpoint in randomized trials comparing AMI therapies. Infarct size is quantified using manual planimetry (MANUAL), visual scoring (VISUAL), or automated techniques using signal-intensity thresholding (AUTO). Although AUTO is considered the most reproducible, prior studies did not account for the subjective determination of endocardial/epicardial borders, which all methods require. For MANUAL and VISUAL, prior studies did not address how to treat intermediate signal intensities due to partial volume.

**Methods:**

To assess sources of variability, AMI size was measured in 30 patients and 12 controls by 3 core-laboratories using 8 methods, each separated by more than 2 months time (*n* = 720 evaluations). The methods were: (1,2) AUTO_Segment_, AUTO_FWHM_ (using Segment software or the full-width-at-half-maximum algorithm, respectively); (3,4) AUTO-UC_Segment_, AUTO-UC_FWHM_ (user correction for endocardial border pixels, no-reflow, etc.); (5) MANUAL; (6) MANUAL-ISI (adjustment for intermediate signal-intensities); (7) VISUAL; (8) VISUAL-ISI.

**Results:**

Mean infarct size varied between 16.8% and 27.2% of LV mass depending on method. Even automated techniques with no user interaction for infarct borders resulted in significant within-patient variability given the need to subjectively trace endocardial/epicardial contours. The coefficient-of-variation (CV) was 10.6% and 14.6% for AUTO_Segment_ and AUTO_FWHM_, respectively. For manual and visual categories, reproducibility was improved when intermediate signal-intensities were considered (MANUAL-ISI vs MANUAL: CV = 8.3% vs 14.4%; *p* = 0.03; VISUAL-ISI vs VISUAL: CV = 8.4% vs 10.9%; *p* = 0.01). For AUTO-UC_Segment_, MANUAL-ISI, and VISUAL-ISI (best technique in each category) within-patient variability due to the quantification method was less than 10% of total variability, and the required sample sizes for detecting a 5% absolute difference in infarct size were 62, 63, and 62 patients, respectively.

**Conclusion:**

Among CMR core-laboratories, an important source of variability in infarct size quantification is the subjective delineation of endocardial/epicardial borders. When intermediate signal intensities are considered in manual planimetry and visual scoring, reproducibility and impact on sample size are similar to automated techniques.

## Background

The ultimate goal in the development of pharmacological therapies for acute myocardial infarction (AMI) is a reduction in mortality. Current treatment strategies in AMI are quite effective, and further reduction in mortality with novel therapies will require increasingly larger sample sizes. The resources associated with large sample sizes limits the number of new therapies that can be tested in clinical trials. Hence, surrogate endpoints of mortality that can assess the efficacy of novel therapies are of interest, and infarct size appears to be particularly attractive given its strong link with outcome. [1, 2] Late gadolinium enhancement (LGE) cardiovascular magnetic resonance (CMR) is considered the imaging reference standard for the assessment of AMI, [2, 3] offering advantages in detecting small and subendocardial infarcts. [4, 5].

Quantification of LGE infarct size can be accomplished by manual planimetry. [6–8] Automated methods, which use the image signal intensity of the infarct and/or normal myocardium to define infarct borders, are believed to be more objective and, therefore, more reproducible. [8–11] However, all automated methods require manual tracing of the LV myocardial contours. This is because there are no automated algorithms that can reliably distinguish the bright LV cavity from the bright endocardial border of the infarct using conventional pulse-sequences, although there is some work attempting to tackle this problem. [12–15] The importance of this component in the overall reproducibility of infarct size quantification is unknown. Prior studies evaluating the reproducibility of methods for infarct quantification reported results only after the step of manually tracing the endocardial/epicardial contours had already been performed. [6–11].

A simple method of infarct size quantification is visual scoring of hyperenhanced tissue on a standard 17-segment model with a 5-point scale for each segment. [2, 5] This method allows rapid assessment of infarct size without the need for planimetry of endocardial/epicardial borders. Previous investigations evaluating the reproducibility of visual and manual planimetry methods did not explicitly define how a user should treat partially bright regions with intermediate signal intensities, which are typically located at the infarct border zone and result from partial volume or other effects. [16].

A limitation in the use of LGE for infarct size quantification in clinical trials is the lack of studies evaluating the reproducibility of infarct size measurements at multiple centers. [17] The aim of the present study was to assess sources of variability among automated, manual, and visual methods in the quantification of AMI size. Unlike prior reports, we (a) compared measurements at 3 separate core laboratories, (b) included the step of tracing endocardial/epicardial borders for a complete assessment of reproducibility (e.g. to assess interobserver variability), and (c) explicitly defined how users should treat intermediate signal intensities for manual and visual methods. Finally, in order to illustrate the significance of the findings in the context of clinical trials, the impact of the findings on sample size was calculated.

## Methods

### Population

Thirty consecutive patients with first ST-elevation AMI were enrolled at three centers (10 patients each) that have provided CMR core-laboratory services (Lund University Hospital, Lund, Sweden; Virginia Commonwealth University Health Systems, Richmond, Virginia; Duke University Medical Center, Durham, North Carolina). All patients met the Universal definition of MI, [18] had angiographic confirmation of coronary disease, and underwent CMR within 7 days of hospital admission for AMI. Patients with known prior myocardial infarction, severe valvular disease, concomitant non-ischemic myocardial disorders, or contraindications for CMR (pacemaker or defibrillator) were excluded. A control group of 12 subjects without known heart disease and with very low probability of developing coronary artery disease over the next ten years (Framingham risk less than 1% for women and 2% for men) were also enrolled. Institutional Review Board approval was received from Duke, Virginia Commonwealth, and Lund Universities, and all patients gave written informed consent.

### CMR protocol

Clinical 1.5-T scanners (Siemens Avanto, Erlangen, Germany; Philips Intera, Best, The Netherlands ) with phased-array receiver coils were used to acquire standard breath-held cine and LGE images according to society guidelines. [19] In brief, cine images were acquired in multiple short-axis (throughout the entire LV) and 3 standard cardiac long-axis views using a balanced steady-state free precession sequence (slice thickness, 6–8 mm; temporal resolution, 35–40 ms; in-plane resolution 1.5–1.7 × 1.4–1.6 mm). LGE was performed using a segmented inversion-recovery gradient-echo sequence (slice thickness, 6–8 mm; in-plane resolution 1.5–1.8 × 1.4–1.6 mm) 10–15 min after contrast administration (gadoversetamide or gadopentetate dimeglumine, 0.15–0.20 mmol/kg) in the identical locations as cine-CMR. Inversion delay time was set to null signal from normal myocardium, and was typically 280–360 ms.

### Study protocol

**Fig. 1:**
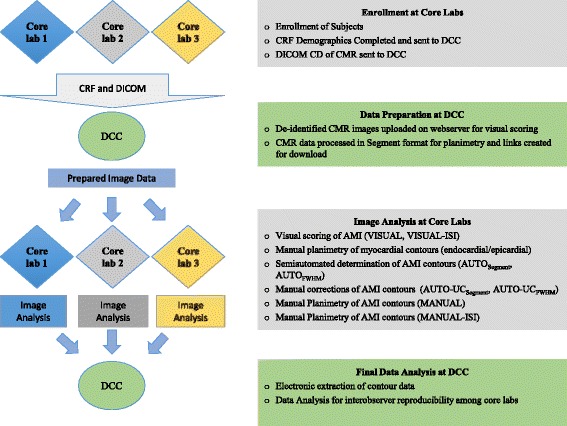
Study Protocol (DCC = data-coordinating center, CRF = case report form)

A standard case report form (CRF) including demographic information, medical history with coronary artery disease (CAD) risk factors, and documentation of AMI was completed for each patient at the participating core-laboratory. The CRFs along with a CD of the CMR scan in DICOM format were submitted to the data-coordinating center (DCC). At the DCC, scans were de-identified, uploaded to a secure web-based PACS system for visual scoring, and processed into the file format for Segment software (v1.9 R2580, Medviso AB, Lund, Sweden) which was used for manual planimetry and automated infarct quantification. [20] All subsequent image data transfers back to and from the individual core-laboratories for the multiple assessments were web-based and electronic (Fig. 1).

### Image analysis

LGE images were analyzed by an experienced reader (HA, JG, IK) at each of the three CMR core-laboratories after completion of group training. A written manual was given to each site with definitions and descriptions of the analysis procedures, and 3 test cases with example measurements were presented at the training session. Infarct size quantification was performed using 8 methods as detailed below. For readers, measurements by different methods were separated in time by >2-months and were performed blinded to the results of prior measurements by the same reader, measurements by other readers, and all clinical data.

For all methods, infarct size was determined on a per patient basis from the stack of short-axis images, however, the long-axis LGE images as well as the cine images were provided to the reader for reference. Prior to quantification, visual perusal of the images was performed to determine if infarction was present or absent. For this step the 12 control subjects without infarction were randomly interspersed among the AMI patients. All 3 readers accurately differentiated AMI patients from controls, hence a total of 720 complete infarct size measurements were performed (30 patients × 8 methods × 3 readers = 720).

#### Automated Infarct Border

**Fig. 2:**
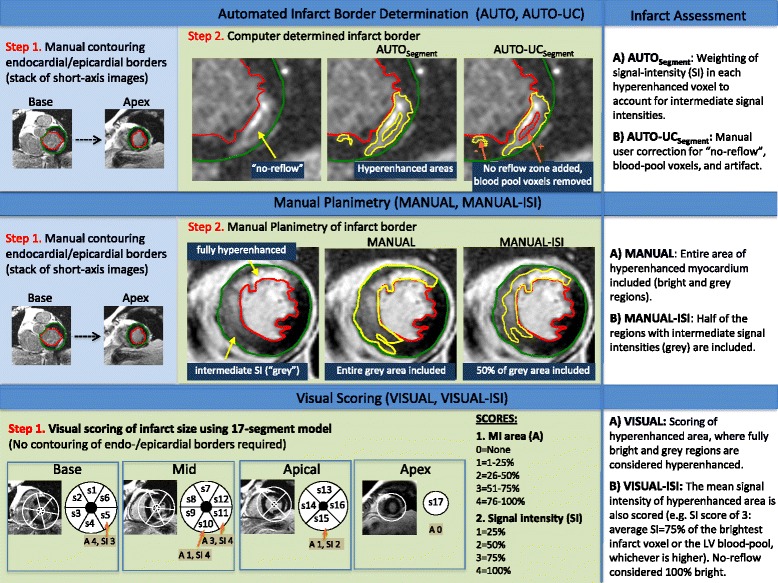
The methods used to quantify infarct size based on late gadolinium enhancement (LGE) are illustrated. The *top row* depicts the steps for automated methods for infarct border determination without (**AUTO**) and with user correction (**AUTO-UC**). Two commonly used techniques for signal thresholding were used, the “Segment”-algorithm (**AUTO**_**Segment**_) and the “full-width at half maximum” (FWHM) technique (**AUTO**_FWHM_). Note that automated methods still require manual delineation of the myocardial (endocardial/epicardial) borders. The middle row depicts the steps for manual planimetry of the infarct. For **MANUAL**, readers were instructed to include any myocardium that appeared hyperenhanced, whether fully bright or partially bright (e.g. *grey*). For **MANUAL-ISI**, adjustments were made for intermediate signal intensities (ISI) in that half of *grey* regions were included (along with 100% of fully bright regions). The *bottom row* depicts visual scoring methods, which were based on the conventional 17-segment model. For **VISUAL**, the spatial extent (area) of hyperenhancement was considered, whereas for **VISUAL-ISI**, the spatial extent and the signal intensity of hyperenhancement were both considered. No-reflow zones were considered fully bright similar to that for MANUAL-ISI. Typical scores in a patient example are shown (A = hyperenhancement area; SI = hyperenhancement signal intensity)

Although the contour of the infarct was determined by a computer algorithm, the first step was to manually contour the LV endocardial/epicardial borders (Fig. 2, top row). Trabeculations and papillary muscles that were completely detached from the myocardial wall were excluded from the myocardium. The infarct border was then determined using the Segment algorithm as described previously (**AUTO**_**Segment**_). [21] In brief, this algorithm accounts for partial volume effects and intermediate signal intensities by assigning a weighting to hyperenhanced voxels depending on the signal intensity, rather than dichotomously classifying voxels as 100% infarcted or normal. Since the computer algorithm was applied without any user input, any within-patient variability in infarct size between the core laboratories for AUTO_**Segment**_ was due to the manual delineation of endocardial/epicardial borders. In other words, even if the infarct border is determined solely by computer there may still be within-patient variability in infarct size measurements, since variability is introduced during manual planimetry of endocardial/epicardial contours.

Myocardial and infarct borders were transmitted to the DCC for quantification. At a separate timepoint, myocardial and infarct borders were transmitted back to the site, and the automatically defined infarct borders were manually corrected for “no-reflow” zones, bright blood-pool or epicardial fat pixels included within the myocardial contour, and artifacts (**AUTO-UC**_**Segment**_). The identical protocol of infarct segmentation was performed using the “full-width at half maximum”-algorithm (FWHM) without (**AUTO**_**FWHM**_) and with manual corrections (**AUTO-UC**_**FWHM**_). [9].

#### Manual Planimetry

The first step again was manual delineation of LV endocardial/epicardial borders. The infarct border was then traced manually in two different ways at separate timepoints. For **MANUAL**, readers were instructed to include any myocardium that appeared hyperenhanced, whether fully bright or partially bright with intermediate signal intensities (e.g. grey). For **MANUAL-ISI**, 100% of fully bright regions along with 50% of intermediate signal intensity (ISI) regions were included (Fig. 2, middle row). For both manual methods, window and level was preset according to society guidelines so that noise was still detectable (nulled myocardium is not a single image intensity) and infarcted regions were not over-saturated (hyperenhanced myocardium is not a single image intensity). [22] Additionally, no-reflow zones were considered 100% infarcted.

#### Visual scoring

Similar to Manual methods, window and level were preset prior to presentation to readers. A standard 17-segment model with a 5-point scale was used to score the spatial extent of hyperenhancement (0 = no hyperenhancement, 1 = 1–25%, 2 = 26–50%, 3 = 51–75%, 4 = 76–100%). For **VISUAL**, both fully bright and partially bright regions were considered hyperenhanced, similar to MANUAL. Infarct size as a percentage of LV myocardium was then calculated by summing the segments with hyperenhancement (each weighted by the midpoint of the range of hyperenhancement for the given segmental score, i.e. 1 = 13%; 2 = 38%; 3 = 63%; 4 = 88%) and dividing by 17. [5] For **VISUAL-ISI**, the mean signal intensity of hyperenhanced myocardium within each segment was also scored relative to the brightest infarct voxel or the LV blood-pool, whichever was higher (Fig. 2, bottom row). This provided adjustment for intermediate signal intensities in the calculation of infarct size, since for each segment the hyperenhancement extent score was weighted by the signal-intensity score prior to summing and then dividing by 17.

#### Signal-to-Noise (SNR) and Contrast-to-Noise Ratio (CNR)

Regions-of-interest were manually drawn in the infarcted region, in remote normal myocardium, and in air (‘noise’). SNR of infarct and normal myocardium was calculated as mean signal intensity of each tissue divided by the SD of noise, respectively. Infarct-to-normal myocardium CNR was calculated by subtracting normal myocardium SNR from infarct SNR.

### Statistical analysis

Data are presented as mean±SD. Infarct size (% LV) was compared between methods using analysis of variance (ANOVA) with repeated measures with Bonferroni correction for multiple post-hoc pairwise comparisons. For each method, reproducibility was assessed by first calculating absolute differences in core-laboratory measurements, averaging these per patient, then calculating the mean and SD of the (averaged) differences for the population. Following this step the coefficient of variation (CV) was determined (defined as the ratio of the SD of the differences divided by the mean value of infarct size as measured by the specific method). [23] To further assess reproducibility the intraclass correlation coefficient (ICC) was also calculated. The significance of differences in reproducibility among methods was evaluated by comparing the SD of the differences using ANOVA with repeated measures. Bland-Altman analyses were performed to assess systematic offsets in measurements between methods and core-laboratories. Linear regression analysis was performed to assess the relation between SNR and CNR measures and the variability of infarct size measurements across core-laboratories. The sample size (*n*) required to detect a potentially important change in infarct size was calculated for each method according to the following formula: [2, 24]

$$ n=\frac{2\left({\sigma}^2\right){\left({z}_{\alpha /2}+{z}_{\beta}\right)}^2}{\delta^2} $$

where δ is the expected reduction in infarct size (3%, 5%, and 7% absolute reduction was used for illustrative purposes), z is the value of the z-statistic with an α = 0.05 and β = 0.2, and σ is the standard deviation of infarct size. The latter is comprised of two components: the variability in infarct size between patients (σ_*T*_), and the variability (σ_*e*_) due to the sizing method (e.g. standard deviation of the differences). All statistical tests were two-sided and *p* < 0.05 was considered statistically significant. The authors had full access to and take full responsibility for the integrity of the data. All authors have read and agree to the manuscript as written.

## Results

### Patient characteristics

Clinical characteristics of the 30 enrolled patients are shown in Table 1. Briefly, the mean age was 57 years, 80% were male, and most (94%) were treated with primary percutaneous revascularization. The infarct-related-artery was the left anterior descending in 43%, the right coronary in 40%, and the left circumflex in 17%.

**Table 1:** Patient characteristics

Characteristic	Entire Group (*n* = 30)
Age (years)	57 ± 11
Male gender	24 (80%)
Vital Parameters	
Systolic Blood Pressure (mmHg)	131 ± 24
Diastolic Blood Pressure (mmHg)	78 ± 17
Height (cm)	173 ± 12
Weight (kg)	85 ± 14
CAD Risk Factors	
Diabetes mellitus	10 (33%)
Hypertension	17 (57%)
Family History of CAD	7 (23%)
Current Smoker	13 (43%)
Hyperlipidemia	14 (47%)
Medications	
Statins	30 (100%)
Beta-blockers	30 (100%)
Aspirin	30 (100%)
Thienopyridine	30 (100%)
ACE-I or ARB	30 (100%)
Peak Troponin	
Troponin T, ng/ml (*n* = 25)	7.3 ± 4.5
Troponin I, ng/ml (*n* = 5)	39.6 ± 18.2
Primary Treatment	
PCI	28 (94%)^a^
Lytics	1 (3%)
None	1 (3%)^b^
Infarct Related Artery	
LAD	13 (43%)
RCA	12 (40%)
LCx	5 (17%)

*CAD* coronary artery disease, *ACE-inhibitor* angiotensin-converting-enzyme inhibitor, *PCI* percutaneous coronary intervention, *LAD* left anterior descending coronary artery, *RCA* right coronary artery, *LCx* left circumflex coronary artery

^a^In 2 patients PCI was not successful

^b^Presented later than 24 h

### Infarct size

Mean infarct size as a percentage of LV myocardial volume for each core-laboratory and overall are shown for the 8 methods in Table 2. Infarct size varied from 16.8% to 27.2% depending on the technique. Adjustment for intermediate signal-intensities resulted in a reduction in infarct size for both manual and visual categories (MANUAL vs MANUAL-ISI, 27.2% vs 19.3%, *p* < 0.001; VISUAL vs VISUAL-ISI, 20.4% vs 16.8%, *p* = 0.012); Differences between MANUAL-ISI and VISUAL-ISI did not reach statistical significance (*p* = 0.39). Likewise, differences between AUTO-UC and AUTO did not reach statistical significance (*p* = 1.0 for both Segment- and FWHM-techniques), since user interactions that could increase infarct size (e.g. including no-reflow zones) were likely offset by user interactions that could decrease infarct size (e.g. excluding blood-pool voxels and/or artifact). Infarct size by AUTO-UC_Segment_ and AUTO-UC_FWHM_ was not significantly different than that provided by MANUAL-ISI (*p* = 1.0 for both). Findings were similar when considering infarct size in grams rather than as a percentage of LV mass (Table 2 **)**.

**Table 2:** Mean infarct size by quantification method

Method		Overall Mean	Core Lab 1	Core Lab 2	Core Lab 3
AUTO_Segment_	% LV	18.5 ± 9.5	17.8 ± 8.1	18.9 ± 10.8	18.7 ± 10.3
AUTO-UC_Segment_	% LV	20.2 ± 10.2	20.6 ± 10.4	20.0 ± 10.2	20.0 ± 10.5
AUTO_FWHM_	% LV	18.8 ± 10.3	18.9 ± 9.8	19.9 ± 11.7	17.6 ± 10.1
AUTO-UC_FWHM_	% LV	20.6 ± 11.4	20.5 ± 11.0	20.5 ± 12.4	20.7 ± 11.4
MANUAL	% LV	27.2 ± 13.0	30.6 ± 14.7	26.8 ± 12.9	24.1 ± 12.3
MANUAL-ISI	% LV	19.3 ± 11.0	19.8 ± 10.9	19.7 ± 11.6	18.4 ± 11.1
VISUAL	% LV	20.4 ± 8.0	18.6 ± 7.4	21.0 ± 8.7	21.5 ± 8.8
VISUAL-ISI	% LV	16.8 ± 7.4	16.3 ± 7.1	17.8 ± 7.9	16.4 ± 7.8
AUTO_Segment_	gram	32.8 ± 22.1	32.4 ± 19.1	35.8 ± 28.1	30.1 ± 20.4
AUTO-UC_Segment_	gram	35.6 ± 24.3	37.2 ± 24.2	37.3 ± 27.9	32.1 ± 22.0
AUTO_FWHM_	gram	33.1 ± 24.8	34.0 ± 24.5	37.2 ± 32.0	28.0 ± 20.3
AUTO-UC_FWHM_	gram	35.6 ± 26.9	36.1 ± 24.8	38.3 ± 34.2	32.3 ± 23.2
MANUAL	gram	49.9 ± 30.6	54.4 ± 36.0	47.1 ± 29.8	42.3 ± 27.5
MANUAL-ISI	gram	34.1 ± 25.6	35.7 ± 24.0	37.1 ± 31.3	29.5 ± 22.7

Values ± standard deviation

AUTO_Segment_ = automated infarct quantification without user correction (Segment)

AUTO-UC_Segment_ = automated infarct quantification with user correction (Segment)

AUTO_FWHM_ = automated infarct quantification without user correction (FWHM)

AUTO-UC_FWHM_ = automated infarct quantification with user correction (FWHM)

MANUAL = manual planimetry including the entire area of hyperenhanced myocardium (all bright and grey regions)

MANUAL-ISI = manual planimetry with adjustment for regions with intermediate signal intensity (all bright and half of grey regions included)

VISUAL = visual scoring of the extent of hyperenhancement (both bright and grey regions are considered hyperenhanced). Data are available only for infarct size as %LV

VISUAL-ISI = visual scoring of the extent of hyperenhancement weighted by the degree of hyperenhancement. Data are available only for infarct size as %LV

There were excellent correlations for infarct size measurements between AUTO-UC_Segment_, AUTO-UC_FWHM_, MANUAL-ISI, and VISUAL-ISI. For the 6 two-way comparisons, the correlation coefficient was 0.90 or higher (*p* < 0.0001 for all six comparisons). For this analysis, the infarct size measurements among the 3 core-laboratories were averaged for each patient prior to comparison.

### Reproducibility and bias

The reproducibility data are summarized in Table 3. Even when infarct borders were delineated in a completely automated fashion by computer, there was significant variability in infarct size among core-laboratories (AUTO_Segment_: CV = 10.6%, AUTO_FWHM_: CV = 14.6%). This was due to the variability in manual delineation of endocardial/epicardial contours, which was a necessary first step. Adding user input to correct computer generated infarct borders resulted in a mild improvement in reproducibility for both Segment and FWHM methods (AUTO-UC_Segment_: CV = 8.3%; AUTO-UC_FWHM_: CV 9.8%), however, the improvement was significant only for Segment (*p* = 0.045). For manual and visual categories, explicitly adjusting for regions with intermediate signal-intensity led to improved reproducibility (MANUAL-ISI vs MANUAL: CV = 8.3% vs 14.4%; *p* = 0.03; VISUAL-ISI vs VISUAL: CV = 8.4% vs 10.9%; *p* = 0.01). When the best technique in each of the 3 categories were compared, reproducibility was similar (AUTO-UC_Segment_, MANUAL-ISI, and VISUAL-ISI: CV = 8.3%, 8.3%, 8.4%, respectively). Findings were similar when infarct size was measured in grams rather than as a percentage of LV mass (Table 3). The reproducibility of the measurement of LV mass itself was moderate (CV = 7.0%, ICC 0.85 [0.77, 0.92]).

**Table 3:** Summary of reproducibility analysis

		CV	ICC
AUTO_Segment_	% LV	10.6%	0.91 [0.86, 0.95]
AUTO-UC_Segment_	% LV	8.3%	0.96 [0.93, 0.98]
AUTO_FWHM_	% LV	14.6%	0.90 [0.85, 0.95]
AUTO-UC_FWHM_	% LV	9.8%	0.94 [0.91, 0.97]
MANUAL	% LV	14.4%	0.87 [0.79, 0.93]
MANUAL-ISI	% LV	8.3%	0.94 [0.90, 0.97]
VISUAL	% LV	10.9%	0.85 [0.77, 0.92]
VISUAL-ISI	% LV	8.4%	0.90 [0.84, 0.95]
AUTO_Segment_	gram	19.2%	0.89 [0.82, 0.95]
AUTO-UC_Segment_	gram	15.9%	0.93 [0.89, 0.96]
AUTO_FWHM_	gram	31.4%	0.83 [0.74, 0.91]
AUTO-UC_FWHM_	gram	24.7%	0.90 [0.84, 0.95]
MANUAL	gram	18.7%	0.90 [0.85, 0.95]
MANUAL-ISI	gram	20.4%	0.90 [0.84, 0.95]

*CV* coefficient of variation

*ICC* intraclass correlation coefficient (values [95% confidence interval])

For VISUAL and VISUAL-ISI, data are available only for infarct size as %LV

**Fig. 3:**
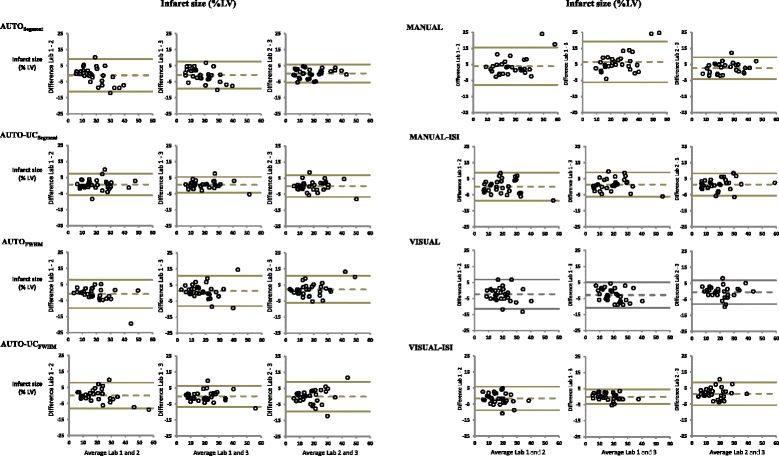
Bland-Altman plots are shown for each of the 3 pairwise core-laboratory comparisons using 8 methods for infarct size quantification. The y-axis represents the difference in infarct size between the two labs in terms of percentage LV myocardium

Bland-Altman plots were made for each of the 3 pairwise core-laboratory comparisons and for each of the 8 methods (Fig. 3). A summary of the findings is shown in Table 4. In general, there were often systematic offsets between core-laboratories, however, for AUTO-UC_Segment_, AUTO-UC_FWHM_, MANUAL-ISI, and VISUAL-ISI, biases were minimal (all ≤1.5%). Additionally, examination of the plots confirmed that differences in infarct size were not related to mean infarct size.

**Table 4:** Bland-Altman analysis of pairwise comparisons between core-laboratories for all methods

	Core-lab 1 vs Core lab 2		Core-lab 1 vs Core lab 3		Core-lab 2 vs Core lab 3	
	Difference ± SD	95% Limits of Agreement	Difference ± SD	95% Limits of Agreement	Difference ± SD	95% Limits of Agreement
AUTO_Segment_	−1.0 ± 5.0 *p* = 0.28	−11.1, 9.0	−0.8 ± 4.2 *p* = 0.29	−9.2, 7.6	0.2 ± 2.8 *p* = 0.72	−5.4, 5.8
AUTO-UC_Segment_	0.6 ± 3.4 *p* = 0.32	−6.1, 7.4	0.6 ± 2.4 *p* = 0.17	−4.3, 5.5	0.0 ± 3.4 *P* = 0.99	−6.8, 6.8
AUTO_FWHM_	−1.0 ± 4.4 *p* = 0.24	−9.7, 7.8	1.3 ± 4.7 *p* = 0.15	−8.1, 10.7	2.2 ± 4.2 0.007	−6.1, 10.7
AUTO-UC_FWHM_	0.03 ± 4.0 *p* = 0.97	−8.0, 8.1	−0.2 ± 3.3 *p* = 0.77	−6.8, 6.4	−0.3 ± 4.6 *p* = 0.81	−9.5, 9.1
MANUAL	3.9 ± 5.9 *p* < 0.01	−7.8, 15.6	6.5 ± 6.4 *p* < 0.01	−6.2, 19.3	2.6 ± 3.4 *p* < 0.01	−4.1, 9.4
MANUAL-ISI	0.0 ± 4.4 *p* = 0.98	−8.7, 8.8	1.4 ± 3.8 *p* = 0.06	−6.2, 9.0	1.4 ± 3.5 *p* = 0.04	−5.5, 8.3
VISUAL	−2.4 ± 4.6 *p* = 0.01	−11.5, 6.8	−2.9 ± 4.0 *p* < 0.01	−10.8, 5.1	−0.5 ± 3.7 *p* = 0.47	−7.9, 6.9
VISUAL-ISI	−1.5 ± 3.7 *p* = 0.03	−8.9, 5.9	−0.1 ± 2.3 *p* = 0.90	−4.6, 4.5	1.4 ± 3.5 *p* = 0.03	−5.7, 8.5

*SD* standard deviation

There was a poor relationship between image SNR and CNR measures and the variability of infarct size measurements for all eight analysis methods. The absolute value of the correlation coefficient was less than 0.4 for all regressions. Specifically, r ranged between −0.04 to −0.39 for CNR of infarct-to-normal myocardium, between 0.34 to −0.20 for SNR of normal myocardium, and between 0.01 to −0.36 for SNR of infarction.

### Sample size considerations

Using standard variance components analysis, we calculated the variance between-patients (σ_*T*_^2^), within-patients (σ_*e*_^2^), and total variance (σ_*T*_^2^ + σ_*e*_^2^). For the best method in each category (AUTO-UC_Segment_, MANUAL-ISI, and VISUAL-ISI), the variance within-patients due to the quantification method was <10% of total variance. Hence, there were minimal differences between these 3 methods in the calculated sample size needed to detect a 3%, 5%, and 7% absolute reduction in acute infarct size (Table 5).

**Table 5:** Sample size needed (per arm) to detect potential therapeutic effect on infarct size

	3% LV^a^	5% LV^a^	7% LV^a^
AUTO-UC_Segment_	168	62	32
MANUAL-ISI	174	63	33
VISUAL-ISI	170	62	32

^a^3%, 5%, and 7% represent an absolute reduction in infarct size as a percentage of left ventricular mass

## Discussion

In this study we found that automated quantification with a computer algorithm, manual planimetry, and visual scoring can have similar reproducibility when used in core-laboratories for infarct size quantification. This is a surprising finding as many consider a computer algorithm objective and therefore more reproducible than the subjective judgment by a human user. Considerable research has been dedicated to developing and evaluating various thresholding algorithms for infarct size quantification. Bondarenko et al. tested the “n-SD” approach, which is based on measuring the mean and standard deviation of signal in normal, noninfarcted myocardium. [8] Amado et al. advocated the full-width at half-maximum (FWHM) technique, which uses the signal intensity of the infarct rather than normal myocardium for finding the appropriate threshold. [9] Heiberg et al. validated an algorithm, which assigns a weighting to each myocardial voxel depending on its signal intensity above a fixed number of standard deviations above remote, and infarct size is calculated by summing weighted volumes rather than dichotomous volumes. [21] In patient studies, manual planimetry by “experienced observers” is often used as the reference standard since pathology is not available. Usually, excellent agreement between the computer algorithm approach and manual planimetry is reported in these studies. [8, 21] On the other hand, Flett et al. compared the reproducibility of infarct size quantification methods, and found the FWHM technique to have superior reproducibility compared with manual planimetry and the n-SD approach. [10] Regarding prior studies, however, it is important to note that none have taken into account the subjective determination of endocardial/epicardial borders, which all methods require as a necessary first step before determining the infarct borders. In the current study, the results show there can be considerable within-patient variability in infarct size measurements even if the infarct border is determined solely by computer, since variability is introduced during the planimetry of endocardial/epicardial contours.

The importance of this finding is that it is necessary to consider the variability in endocardial/epicardial borders for a thorough comparison between quantification methods and for an accurate calculation of sample size in a clinical trial, since it is a substantial portion of the variability in reproducing measurements. Not surprisingly, Flett et al. in AMI patients reported intraclass correlation coefficients ranging from approximately 0.94 to 0.99 based on predrawn endocardial/epicardial contours, [10] whereas in the current study ICCs were lower, ranging from 0.85 to 0.96. The appreciable variability in endocardial/epicardial borders also highlights that there may be an upper limit in improving reproducibility by means of computer algorithms, and suggests that moderate differences in reproducibility between analysis methods may have limited practical significance, if these differences are based on predrawn LV myocardial contours.

One could try to avoid the variability introduced by subjective planimetry of LV myocardial borders by expressing infarct size in terms of absolute mass (ie. numerator alone) rather than as a percentage of LV myocardial mass (ie. numerator/denominator ratio). This would, however, introduce the variability in heart size. Furthermore, this is unlikely to succeed in the setting of a subendocardial MI since the endocardial border of the infarct is almost always the same as the local LV myocardial − bloodpool border. Hence, this portion of the infarct contour will be the result of manual planimetry even if a computer algorithm is used to determine the infarct borders. In the setting of a transmural MI, both the endocardial and epicardial aspects of the infarct will result from manual planimetry. Another theoretical approach to reduce variability would be to use an automated method to determine LV myocardial borders on LGE images. However, we are not aware of any automated tool that is publicly available, [12–15] and any attempt to develop such a method will likely be troubled by the fact that both infarction and LV blood-pool are bright and have similar signal intensities. [25] Because the endocardial border of the infarct displays the smallest gradient in image intensities, and can constitute up to 50% of the infarct perimeter, this portion of the infarct border is likely the largest source of variability in infarct size measurements.

To our knowledge, the present study is the first to explicitly define how myocardial regions with intermediate signal-intensity should be considered for quantitative infarct size measurement by manual planimetry or visual scoring. Without explicit instruction, readers might include all, include part, or exclude such regions as part of the infarct. In the current study we tested two approaches (see Fig. 2): (a) to include all regions with intermediate signal-intensity, and (b) to include an adjusted percentage of regions with intermediate signal-intensity. The observation that the two approaches lead to appreciable differences in infarct size for both manual planimetry and visual scoring (e.g. MANUAL-ISI vs MANUAL and VISUAL-ISI vs VISUAL) indicates the spatial extent of these regions can be substantial. It also suggests that without explicit instruction, reader inconsistency in interpreting regions with intermediate signal-intensity, could in part, explain some of the variability that has been found previously with non-automated methods.

Interestingly, explicit instructions to include an adjusted percentage of regions with intermediate signal-intensity, rather than all regions with intermediate signal-intensity, improved the reproducibility of infarct size measurements for manual planimetry and visual scoring. The reason for this not clear, however, it is possible that incorporating a process to “weight” regions with intermediate signal-intensity, may provide a self-correcting mechanism for some of the more idiosyncratic subjective assessments of infarct size.

Similarly, it may seem paradoxical that incorporating subjective user input with AUTO could improve reproducibility compared with excluding user input (AUTO-UC_Segment_ versus AUTO_Segment_: ICC, 0.96 vs 0.91; CV, 8.3% vs 10.6%). However, regarding this point recall that AUTO includes the variability introduced during manual planimetry of endocardial/epicardial borders. Hence imprecise endocardial contours may lead to bright LV cavity blood-pool or epicardial fat pixels mistakenly included as part of quantitative infarct size, even when remote from the infarct zone. In this situation, allowing user input could reduce variability in infarct size measurements since users could “self-correct” for obvious imperfections in the endocardial/epicardial contours. We note that this process of user correction of the endocardial/epicardial contours reflects the actual process by which infarct size quantification commonly is performed in core-laboratories. Hence AUTO-UC (with any thresholding technique used) most closely reflects the standard process, and differences between AUTO-UC and AUTO, provide a quantitative assessment of the user correction step.

In the setting of an acute MI trial, it would be difficult to obtain a baseline MRI before treatment. Hence, infarct size measurements cannot be compared before and after therapy, and efficacy will be based only on an unpaired analysis of the MRI after therapy. In the current study, the best method in each category had excellent and similar reproducibility (AUTO-UC_Segment_, MANUAL-ISI, and VISUAL-ISI: CV = 8.3%, 8.3%, and 8.4%, respectively). Moreover, for these three methods, the within-patient variability due to the method was less than 10% of total variability. In other words, the inherent variability in infarct size in a STEMI cohort—the between-patient variability—was far larger than the variability due to the analysis method. The consequence was minimal differences in sample size calculations among the 3 optimized methods (see Table 5). This finding suggests that if performed in a trained core-laboratory, and explicit instructions are given to account for intermediate signal-intensities, manual planimetry and visual scoring may have comparable reproducibility to an automated technique.

### Study limitations

In this study there is no pathology-based reference standard for infarct size. However, the primary aim of the study was to examine the reproducibility of methods for infarct size quantification, which is highly relevant for clinical trials using CMR infarct size as a surrogate endpoint. We tested only two computer algorithms for the automated approach (Segment and FWHM). Previous investigations have compared the reproducibility of various infarct contouring algorithms, [10, 21] and our goal was not to confirm these findings. Instead, we aimed to evaluate the variability introduced by manual planimetry of LV endocardial/epicardial borders, for which there are no prior data. Since this is a required first step before the application of any automated algorithm, it is independent of the specific algorithm and is a relevant component for accurate sample size calculations. Visual identification of regions with intermediate signal-intensities requires an experienced reader, and even then is subjective. However, the current study was designed to simulate the ‘real-life’ situation of a CMR core-laboratory for a clinical trial, which typically involves experienced readers, and for which many steps are ultimately subjective. Moreover, the main goal was to show that despite some variability associated with this subjective step, if explicit instructions are provided, the variability of a subjective approach can be reduced to a level so that it no longer significantly impacts on sample size calculations. That said, it is important to highlight that our results are based on experienced readers, group completion of training sessions prior to performing measurements, and specific protocols on two scanner platforms and with one particular sequence for LGE. Findings should not be extrapolated outside this scenario, and it is possible that an untrained reader will have more reproducible infarct size measurements with an automated algorithm than with manual planimetry or visual scoring. Finally, the sample sizes reported in Table 5 should be treated with caution, since they are highly dependent on the standard deviation of infarct size in the enrolled population.

## Conclusions

In summary, our results show that an important source of variability in infarct size quantification is the manual delineation of endocardial/epicardial borders. Hence, this component should be included in any comparisons among analysis methods. When regions with intermediate signal-intensities are explicitly considered in manual planimetry and visual scoring, reproducibility and impact on sample size are similar to automated techniques. These results were achieved by applying prespecified protocols and were obtained at CMR core-laboratories by experienced readers after completion of organized training sessions.

## Abbreviations

AMI: Acute myocardial infarction
ANOVA: Analysis of variance
CAD: Coronary artery disease
CMR: Cardiovascular magnetic resonance
CRF: Case report form
CV: Coefficient of variation
DCC: Data coordinating center
ICC: Intraclass correlation
ISI: Intermediate signal intensity
LG: Late gadolinium
LV: Left ventricle
